# The Teratogenic Effects of Prenatal Ethanol Exposure Are Exacerbated by Sonic Hedgehog or Gli2 Haploinsufficiency in the Mouse

**DOI:** 10.1371/journal.pone.0089448

**Published:** 2014-02-19

**Authors:** Henry W. Kietzman, Joshua L. Everson, Kathleen K. Sulik, Robert J. Lipinski

**Affiliations:** 1 The Bowles Center for Alcohol Studies, University of North Carolina at Chapel Hill, Chapel Hill, North Carolina, United States of America; 2 Department of Comparative Biosciences, University of Wisconsin-Madison, Madison, Wisconsin, United States of America; Xavier Bichat Medical School, INSERM-CNRS - Université Paris Diderot, France

## Abstract

Disruption of the Hedgehog signaling pathway has been implicated as an important molecular mechanism in the pathogenesis of fetal alcohol syndrome. In severe cases, the abnormalities of the face and brain that result from prenatal ethanol exposure fall within the spectrum of holoprosencephaly. Single allele mutations in the Hh pathway genes *Sonic Hedgehog* (*SHH*) and *GLI2* cause holoprosencephaly with extremely variable phenotypic penetrance in humans. Here, we tested whether mutations in these genes alter the frequency or severity of ethanol-induced dysmorphology in a mouse model. Timed pregnancies were established by mating *Shh^+/−^* or *Gli2^+/−^* male mice backcrossed to C57BL/6J strain, with wildtype females. On gestational day 7, dams were treated with two ip doses of 2.9 g/kg ethanol (or vehicle alone), administered four hrs apart. Fetuses were then genotyped and imaged, and the severity of facial dysmorphology was assessed. Following ethanol exposure, mean dysmorphology scores were increased by 3.2- and 6.6-fold in *Shh^+/−^* and *Gli2^+/−^* groups, respectively, relative to their wildtype littermates. Importantly, a cohort of heterozygous fetuses exhibited phenotypes not typically produced in this model but associated with severe holoprosencephaly, including exencephaly, median cleft lip, otocephaly, and proboscis. As expected, a correlation between the severity of facial dysmorphology and medial forebrain deficiency was observed in affected animals. While *Shh^+/−^* and *Gli2^+/−^* mice have been described as phenotypically normal, these results illustrate a functional haploinsufficiency of both genes in combination with ethanol exposure. By demonstrating an interaction between specific genetic and environmental risk factors, this study provides important insights into the multifactorial etiology and complex pathogenesis of fetal alcohol syndrome and holoprosencephaly.

## Introduction

Holoprosencephaly (HPE) occurs in approximately 1 in 10,000 live births [1], [2] but an observed prevalence of 1 in 250 conceptuses argues that it is one of the most common human developmental abnormalities [3]. Defined by incomplete division of the forebrain and characterized by medial forebrain deficiencies, HPE frequently co-occurs with facial abnormalities, including clefts of the lip and/or palate, microphthalmia, hypotelorism, and midfacial hypoplasia [4]. Notably, in both humans and animal models, these facial phenotypes as well as medial forebrain deficiencies can result from prenatal ethanol exposure [5]–[7]

The Hedgehog (Hh) signaling pathway is required for midline development of the brain and face [8]–[10]. *Sonic Hedgehog* (*Shh*) expression in the neuroectoderm of the diencephalon is critical for ventral patterning and expansion of the medial forebrain [11], [12]. A subsequently established parallel field of *Shh* expression in the surface ectoderm regulates growth of the adjacently developing midface [9], [13]. Genetic and chemical lesions in the Hh signaling pathway have been shown to cause the characteristic face and brain abnormalities of HPE [14]–[18]. While multiple mechanisms have been proposed, disruption of the Hh signaling pathway has also been implicated in the genesis of fetal alcohol syndrome (FAS) [19]–[23].

Mutations in *SHH* are the most commonly identified cause of non-chromosomal HPE, accounting for approximately 12% of such cases [24]–[26]. Mutations in the GLI-Kruppel family member *GLI2*, which encodes a zinc finger protein that serves as the dominant transcriptional activator of the pathway, have also been associated with HPE [27]. However, even in cases with a known causative gene, HPE is etiologically complex. For example, in a recent analysis of 396 individuals representing 157 unrelated kindreds with *SHH* mutations, only 36% were found to have true HPE [25]. The majority of mutation carriers were classified as unaffected or as having microform HPE (i.e. midline facial abnormalities in the absence of detectable neuroanatomical anomalies). This suggests that gene-gene or gene-environment interactions are operational in the pathogenesis of HPE [28].

*Shh^−/−^* mice exhibit severe HPE phenotypes, including a single telencephalic vesicle and proboscis situated above a single central eye [8]. *Gli2^−/−^* mice fail to develop a floor plate and present with microcephaly, cleft palate, and maxillary and mandibular hypoplasia [29], [30]. In contrast, relative to their wildtype littermates, *Shh^+/−^* and *Gli2^+/−^* mice are phenotypically unremarkable [27], [30]. These animals therefore serve as an ideal model in which to test the influence of environmental factors in the context of human disease-relevant genetic predisposition. Such interactions have been proposed as the basis for numerous complex diseases but identification of specific interacting factors has proven difficult. By demonstrating a functional gene-environment interaction between mutations in *Shh* and *Gli2* and prenatal ethanol exposure, the study described here provides new insights into potential mechanisms contributing to the etiology and pathogenesis of FAS and HPE.

## Materials and Methods

### Animals and timed matings

This study was carried out in strict accordance with the recommendations in the Guide for the Care and Use of Laboratory Animals of the National Institutes of Health. All procedures involving animals were approved by the University of North Carolina at Chapel Hill Institutional Animal Care and Use Committee (protocol number 13-081.0). C57BL/6J wildtype female mice were purchased from The Jackson Laboratory (Bar Harbor, ME). *Shh^+/−^* and *Gli2* ^+/*−*^ transgenic mice [8], [31] were backcrossed to the C57BL/6J background for more than ten generations. Two female mice were placed with a single *Shh* or *Gli2* heterozygous male for 2 hrs early in the light cycle and subsequently examined for the presence of copulation plugs, marking gestational day (GD)0. Genotyping was performed as described for *Gli2* mice [30] and using a standard genotyping protocol, “NEOTD,” provided by the Jackson Laboratories and available at http://jaxmice.jax.org for *Shh* mice.

### Ethanol exposure

Timed-pregnant mice were administered two 25% ethanol (v/v in lactated Ringer’s solution) dosages of 2.9 g/kg by ip injection four hrs apart beginning at GD7 [6], [32], [33]. Vehicle treated mice received two volume-equivalent doses of Ringer’s solution.

### Dissection and imaging

On GD17, pregnant dams were euthanized via CO_2_ anesthetization, followed by cervical dislocation. Fetuses were then fixed in formalin (10% in phosphate buffered saline solution) for at least two weeks and then photographed. To ensure consistent orientation, each fetus was stabilized in a wax mold with specific anatomical features of the head carefully aligned to a grid of vertical and horizontal crosshairs. Subsequently, brains were removed from a subset of fetuses by dissection. Images were captured with a MicroPubisher 5.0 camera using QCapture Suite Software.

### Assessment of facial dysmorphology

**Figure 1:**
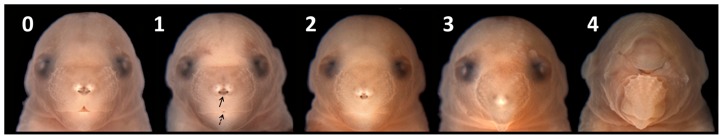
Facial dysmorphology rating scale. Illustrated are a GD 17 fetus having normal facial morphology and 4 fetuses with varying degrees of medial facial deficiency. Numbers assigned to each image (0–4) are scores representing differing degrees of severity of facial dysmorphology. As compared to normal fetuses, those receiving a score of 1 had a notably diminished area of pigmentation between the nostrils (solid arrow) accompanied by reduction in the depth of the normally present median central notch of the upper lip (dashed arrow). A score of 2 was assigned to fetuses that had lost the median lip notch, but still had some remaining pigment at the tip of the nose. Individuals presenting with a single central nostril were assigned a score of 3 and those given a score of 4 had no nostrils.

For unbiased assessment of facial dysmorphology, a semi-quantitative scale encompassing the range of severity present in the study population was established (Fig. 1). Based upon a large reference population of untreated wildtype C57BL/6J GD17 fetuses, a score of 0 was assigned to apparently normal individuals. Scores of 1–4 were assigned based on visual assessment of degree of medial facial deficiency as evidenced by internasal distance and upper lip morphology. Those fetuses receiving a score of 1 had a notably diminished area of pigmentation between the nostrils (Fig. 1, solid arrow) accompanied by reduction in the depth of the normally present median central notch of the upper lip (Fig. 1, dashed arrow). A score of 2 was assigned to those fetuses that had lost the median lip notch, but still had some remaining pigment at the tip of the nose. Individuals presenting with a single central nostril were assigned a score of 3 and those given a score of 4 had no nostrils. Animals having a median cleft lip were classified based on their nasal appearance. All images were examined by a single rater blinded to treatment and genotype.

## Results

*Shh^+/−^* or *Gli2^+/−^* male mice backcrossed to the C57BL/6J background were mated with wildtype females, facilitating direct comparison between littermates differing only in gene dosage. To examine whether these normally silent mutations alter the frequency or severity of ethanol-induced abnormalities, a well characterized prenatal ethanol exposure paradigm that recapitulates the salient facial features of FAS in wildtype C57BL/6J mice was employed. As previously described, considerable intra- and interlitter variability was observed among ethanol-exposed fetuses, with phenotypes ranging from apparently normal to severely dysmorphic [6]. A semi-quantitative scale based upon the degree of medial facial deficiency was then applied to characterize the severity of facial dysmorphology across the entire study population (Fig. 1).

**Figure 2:**
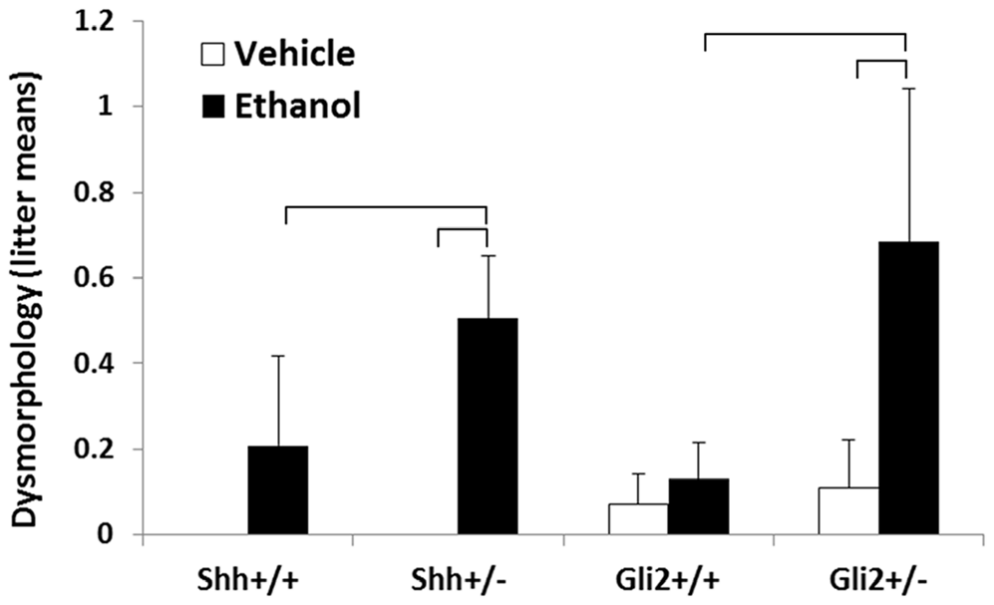
Effect of treatment and genotype on facial dysmorphology. To avoid litter bias, the average dysmorphology score from each genotypic group was determined for each litter in the study population. Values represent the mean plus the standard error of litter averages for each genotype and treatment. Brackets indicate p values of ≤ 0.05 as determined by a one-tailed student’s t-test.

The distribution of facial dysmorphology by treatment and genotype is shown in Table 1. While ethanol exposure resulted in facial dysmorphology meeting the criteria of the implemented scale only rarely in the wildtype cohort, fetuses having defects representative of the full range of the spectrum of severity were frequently observed in the *Shh^+/−^* and *Gli2^+/−^* groups. Comparison of the values listed in table 1 illustrates that ethanol exposure resulted in mean dysmorphology scores that were increased by 3.2 and 6.6 fold in *Shh^+/−^* and *Gli2^+/−^* fetuses, respectively, compared to wildtype littermates. To assess statistical differences between groups independent of litter bias, mean dysmorphology scores with litter averages of genotypic cohorts as the unit of measurement were also calculated (Fig. 2). In the *Shh^+/−^* and *Gli2^+/−^* groups, ethanol exposure caused a significant increase in mean dysmorphology scores relative to both respective vehicle control groups, as well as ethanol-exposed wildtype groups.

**Table 1:** Facial dysmorphology scores by treatment and genotype.

Treatment	Genotype	Sample	Distribution of facial dysmorphology						
		Size	0	1	2	3	4	Sum	Mean
**Vehicle**	*Shh^+/+^*	22	22	0	0	0	0	0	**0**
	*Shh^+/−^*	25	25	0	0	0	0	0	**0**
	*Gli2^+/+^*	34	32	2	0	0	0	2	**0.06**
	*Gli2^+/−^*	17	16	0	1	0	0	2	**0.12**
**Ethanol**	*Shh^+/+^*	28	26	1	0	0	1	5	**0.18**
	*Shh^+/−^*	47	34	4	5	3	1	27	**0.57**
	*Gli2^+/+^*	31	29	0	2	0	0	4	**0.13**
	*Gli2^+/−^*	21	12	4	2	2	1	18	**0.86**

**Figure 3:**
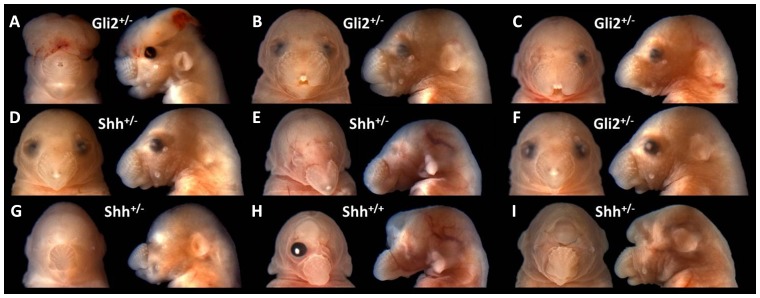
Subpopulation of GD17 fetuses exhibiting severe craniofacial phenotypes. Included in the study population were 9 fetuses with phenotypes not typically observed in wildtype C57BL/6J mice exposed to the employed ethanol exposure paradigm (A-I). Single allele mutations in *Shh* or *Gli2* were detected in 8 of 9 fetuses in this severely affected subpopulation. In addition to varying degrees of upper midfacial deficiency, other notable defects included exencephaly (A), iridial coloboma and microphthalmia (A-D), apparent anophthalmia (E, G, I), agnathia (E), micrognathia (A-D, F-I), and proboscis (I). Median cleft lip was also observed (B, C). Within this subpopulation, fetuses were assigned dysmorphology scores as follows: 2 (A), 3 (B-F), 4 (G-I).

The employed ethanol exposure paradigm is well characterized and has been utilized in numerous studies conducted by the authors of this study [5], [6], [32]–[34]. Importantly, a relatively large cohort of fetuses in the population described here exhibited facial abnormalities not commonly observed in affected wildtype C57BL/6J mice. Along with varying degrees of upper midfacial deficiency, fetuses in this subpopulation exhibited exencephaly, apparent anophthalmia, agnathia, and apparent proboscis (Fig. 3). Genotyping revealed that 8 of 9 of fetuses in this subpopulation with severe phenotypes were either *Shh* or *Gli2* heterozygotes.

**Figure 4:**
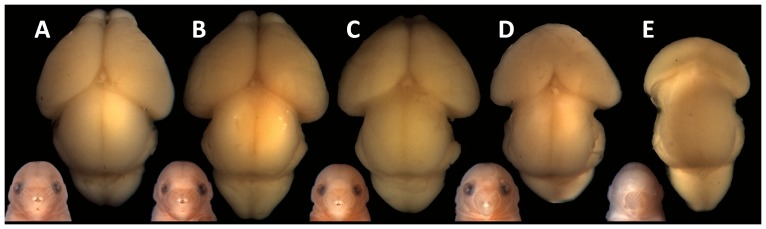
Facial dysmorphology predicts medial forebrain deficiency. Superior views of dissected brains are shown for a normal fetus (A) and for representative examples of each category of facial dysmorphology (B-E). Medial facial deficiency was associated with increasing hypoplasia of the cerebral cortices (B-E), increasing hypoplasia (B, C) or absence of the olfactory bulbs (D, E), and incomplete division of the forebrain (D, E).

In studies of both animal models and human populations, the severity of FAS and HPE- associated facial dysmorphology generally corresponds to that of the brain [33], [35]. As expected, the degree of medial forebrain deficiency directly correlated with increasing category of facial dysmorphology among fetuses in this study population (Fig. 4). True HPE, as defined by incomplete division of the forebrain, was observed in fetuses in the most severe upper midfacial deficiency categories.

## Discussion

While *Shh^+/−^* and *Gli2^+/−^* mice have no apparent phenotype, single allele mutations of these genes in human populations cause HPE with incomplete penetrance or variable expressivity. The findings of the study reported here illustrate that in the mouse, both *Shh* and *Gli2* are functionally haploinsufficient when combined with prenatal ethanol exposure. While mice utilized in biomedical research are maintained in a highly controlled environment, human populations are subject to myriad environmental influences, potentially including ethanol exposure. Thus, demonstration that these genetic lesions lend a predisposition to a near-ubiquitous environmental influence offers new insight into the apparent discrepancy between findings from mouse models versus human populations.

This study follows recent work demonstrating that mutations in *Cdon* exacerbate the effects of prenatal ethanol exposure, producing severe HPE phenotypes in mice [36]. While primarily studied as a co-receptor for the Shh ligand, Cdon is known to be multifunctional with some Hh-independent activity. Promiscuity of receptor activity left the authors to speculate that the observed interaction between Cdon mutation and ethanol exposure may be mediated through effects on the Nodal or BMP signaling pathways, which have also been implicated in the pathogenesis of HPE [37], [38]. By directly examining the interaction between mutations in two genes essential for signal transduction, the findings reported here strongly support the premise that Hh signaling-related genetic lesions directly lend a functional predisposition to the effects of prenatal ethanol exposure.

These findings support the premise that a lower threshold of ethanol exposure is sufficient to cause clinically significant abnormalities in fetuses with genetic mutations in the Hh signaling pathway. However, each of these studies employed a binge model of early prenatal ethanol exposure, which has been reported to result in peak blood ethanol concentrations above 0.04 g/dl [6]. Translation of these findings would benefit by future studies examining the dose-response relationship of ethanol exposure in models of relevant genetic predisposition.

The severity of abnormalities in children exposed prenatally to ethanol appears to depend upon variables beyond the level of exposure itself [39], [40]. Animal studies have confirmed that the teratogenic effects of ethanol vary depending upon genetic background [22], [41]–[44] spurring efforts directed at identifying genetic factors that may predispose the fetus to ethanol teratogenicity [45], [46]. In this regard the results of the present study, along with those of others, strongly argue that additional research examining genetic lesions in the Hh pathway is warranted. Designed to isolate the impact of heterozygosity on embryonic sensitivity to ethanol exposure, for this study *Shh* and *Gli2* null alleles were backcrossed to the C57BL/6J background and heterozygous males were mated with wildtype females. Examination of potential maternal effects of these mutations and whether the demonstrated haploinsufficiency is affected by background strain was not within the scope of this study but would be an interesting future direction as well.

Most diseases result from an interaction of genetic and environmental influences but identification of specific interacting influences has remained largely elusive. The significance of the findings presented here is highlighted by the clinical relevance of the identified interacting factors. Mutations in *SHH* are the most commonly identified cause of non-chromosomal HPE, while it is estimated that 7.6% of women in the United States consume ethanol while pregnant [47]. By demonstrating an interaction between prenatal ethanol exposure and genes in the Hh pathway, this study provides important insights into the multifactorial etiology and pathogenesis of both FAS and HPE.
